# The Anti-Addiction Drug Ibogaine and the Heart: A Delicate Relation

**DOI:** 10.3390/molecules20022208

**Published:** 2015-01-29

**Authors:** Xaver Koenig, Karlheinz Hilber

**Affiliations:** Department of Neurophysiology and Neuropharmacology, Center for Physiology and Pharmacology, Medical University of Vienna, Schwarzspanierstrasse 17, Vienna 1090, Austria

**Keywords:** anti-addiction therapy, cardiotoxicity, drug-induced long QT, hERG channel inhibition, ibogaine, indole alkaloid, 18-methoxycoronaridine, noribogaine, Torsade de Pointes arrhythmias

## Abstract

The plant indole alkaloid ibogaine has shown promising anti-addictive properties in animal studies. Ibogaine is also anti-addictive in humans as the drug alleviates drug craving and impedes relapse of drug use. Although not licensed as therapeutic drug and despite safety concerns, ibogaine is currently used as an anti-addiction medication in alternative medicine in dozens of clinics worldwide. In recent years, alarming reports of life-threatening complications and sudden death cases, temporally associated with the administration of ibogaine, have been accumulating. These adverse reactions were hypothesised to be associated with ibogaine’s propensity to induce cardiac arrhythmias. The aim of this review is to recapitulate the current knowledge about ibogaine’s effects on the heart and the cardiovascular system, and to assess the cardiac risks associated with the use of this drug in anti- addiction therapy. The actions of 18-methoxycoronaridine (18-MC), a less toxic ibogaine congener with anti-addictive properties, are also considered.

## 1. Introduction

Ibogaine is a naturally occurring, psychoactive indole alkaloid derived from the root bark of the African shrub *Tabernanthe iboga*. In West Central Afrika, low dosages of *Tabernanthe iboga* extracts have been employed by indigenous people against fatigue, hunger and thirst. Higher dosages are used for initiation rituals during religious ceremonies. Ibogaine ingestion can lead to intense visions with closed eyes reminding of a waking dream, often accompanied by a vivid recall of autobiographical visual memories [1]. The mechanisms by which ibogaine exerts its psychoactive effects in the brain are only poorly understood, which is attributable to the alkaloid’s complex pharmacology. Effects on multiple neurotransmitter systems via numerous brain targets have been reported. Among those, ibogaine interacts with neurotransmitter transporters, opioid receptors, sigma receptors, glutamate receptors, and nicotinic receptors in low micromolar concentrations [2,3,4,5,6]. Long-lasting effects after ibogaine intake are attributed to the alkaloid’s long-lived active metabolite noribogaine [7,8,9].

Ibogaine’s medical history in the Western world began in the early 1900s when the substance was indicated for the treatment of asthenia and as a neuromuscular stimulant [2]. In the 1940s and 1950s, ibogaine’s suitability as potential cardiovascular drug was studied (e.g., [10]). Later in the 1960s, the drug received much attention because of its potential applicability as an anti-addiction medication. In animal studies, ibogaine has shown promising anti-addictive properties [1]. Thus, ibogaine-treated rodents exhibit attenuated opioid withdrawal symptoms, and diminished self-administration of a variety of drugs of abuse including opioids, cocaine, nicotine, and alcohol [2,3]. Anecdotal evidence suggests that ibogaine is also anti-addictive in humans. When ibogaine is administered to treat drug dependence, typically as single one-time dose [2], patients commonly report sustained resolution of the withdrawal syndromes within 12–18 h, and a reduction in drug craving for prolonged time periods up to several weeks [6]. In 1993, the U.S. Food and Drug Administration (FDA) approved a clinical trial in humans to study the effects of ibogaine. The sudden death of a female patient, however, dampened further interest, and the National Institute on Drug Abuse (NIDA), critically advised by pharmaceutical industry consultants, opted not to fund additional human studies in 1995. Ibogaine was stigmatized as hallucinogen and stimulant with a possible abuse potential, and consequently classified as a Schedule 1 substance in the U.S. [6]. Mostly because of ibogaine’s status as a banned substance in the U.S., the development of the drug’s use in addiction treatment took then place outside conventional clinical and medical settings. The great and rapidly growing popularity of ibogaine as anti-addiction medication in alternative medicine prompted Frank Vocci, former director of NIDA’s anti-addiction drug development, to term ibogaine therapy “a vast, uncontrolled experiment” [11]. Since that time the ibogaine “medical subculture” has continued to expand [6], and today, the alkaloid is used as an anti-addiction medication in alternative medicine in dozens of clinics worldwide [1,6]. Although, as described above, all the efforts to clinically approve ibogaine have failed as yet, NIDA has recently committed financial support for preclinical testing and chemical manufacturing, as well as control work intended to enable clinical trials to develop the synthetic ibogaine congener 18-methoxycoronaridine (18-MC) as a pharmacotherapy for addiction [12,13]. 18-MC also exhibits anti-addictive effects, and is less toxic in animals than ibogaine [4,14,15,16,17].

Ibogaine’s complex pharmacology entails a considerable potential to generate adverse effects. Besides neurotoxic actions (e.g., [2,5,18,19,20]), ibogaine also affects the cardiovascular system, and, in recent years, alarming reports of life- threatening complications and sudden death cases, temporally associated with the administration of the alkaloid, have been accumulating (Table 1). It was hypothesised that the above-mentioned sudden deaths cases in humans were related to cardiac arrhythmias. These are most probably associated with ibogaine’s propensity to induce a QT interval prolongation in the electrocardiogram (ECG) [1,6,21], which is known to enhance the risk for life-threatening Torsade de pointes (TdP) arrhythmia generation [22,23]. The aim of this review is to summarize and discuss the current knowledge about ibogaine’s effects on the heart and the cardiovascular system. The effects of 18-MC are also considered. We further list recent case reports of long QT, arrhythmias, and sudden cardiac death in association with the intake of ibogaine. At the end we discuss practical impacts for the future application of ibogaine and 18-MC as anti-addiction drugs.

## 2. Chemistry and Pharmacokinetics

Alkaloids can be classified on the basis of their underlying chemical structure, the aromatic heterocyclic indole is thus, e.g., the eponym for the indole alkaloids family. Other classes include the pyrrolidines, pyridines, tropanes, pyrrolizidines, isoquinolines, quinolines, terpenoids, and the steroids. Indole alkaloids have been found in a variety of species including fungi, plants and animals. Most famous representatives include the mushroom hallucinogens psilocin and psilocybin, the ergot alkaloids, as well as the pesticide strychnine. Of medical relevance are other indole alkaloids occurring in plants, e.g., reserpine, yohimbine, and ajmaline.

**Figure 1:**
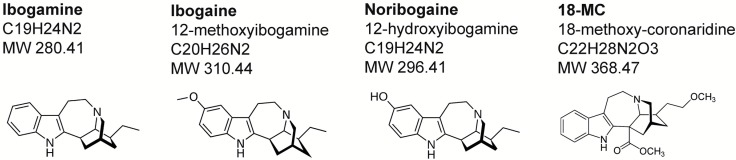
Strucures of ibogamine, ibogaine, noribogaine, and 18-MC with respective full names, chemical formula, and molecular weight (MW).

Alternatively, alkaloids can be classified according to their biological occurrence. As such, ibogaine belongs to the iboga alkaloids, a family of about 80 structurally closely related monoterpene indole alkaloids that can be found in plants belonging to the family of *Apocynacae*, including *Tabernanthe iboga*, *Voacanga africana* and *Tabernaemontana undulata*. The typical characteristics for iboga alkaloids include a tri-cyclic framework harbouring discernible 2-ethylindolyl and isoquinuclidinyl moieties, and a bridgehead nitrogen (Figure 1).

The parent substance is ibogamine, with the systematic name (IUPAC): [6*R*-(6α,6aβ,7β,9α)]-7-ethyl-6,6a,7,8,9,10,12,13-octahydro-6,9-methano-5*H*-pyrido-[1',2':1,2]-azepino-[4,5-b]-indole. The addition of methoxy and hydroxy groups at the 12-position yields the closely related ibogaine (12-methoxyibogamine) and noribogaine (12-hydroxyibogamine), respectively. 18-Methoxycoronaridine (18-MC) is a synthetic congener of ibogaine, which is based on a coronaridine framework (Figure 1).

**Table 1:** Case reports of ibogaine- associated fatalities, long QT, and cardiac arrhythmias.

Age (years)	Sex	Year of Publication	Time after Drug Intake	Diagnosis	Dosage (mg/kg)	Ibogaine (blood, mg/L) ^$^	Electrolyte Levels	QT_c_ (ms)	History/Pathology other Intoxication	Ref.
24–54	m+f	1990–2007	1.5–76 h	sudden death	4.5–29.0	0.24–9.3	na	na	cardiovascular diseases, hepatitis, liver cirrhosis, opiates, cocaine, alcohol, diazepam	[1]
52	m	2013	12–24 h	sudden death	na	2	low K, Mg ^#^	na	alcoholism, atherosclerosis, liver cirrhosis na	[24]
27	m	2013	12 h	sudden death	1.5–2.2	0.65–1.27	na	na	multiple substance addiction, no cardiac pathology no methadone >48 h, diazepam	[25]
25	m	2013	48 h	sudden death	6.25 *	na	na	na	heroin addiction, supraventricular tachycardia na	[26]
31	f	2009		long QT, VT	8.75 *	na	low K, Mg	616	alcohol addiction no alcohol, no other drugs	[21]
49	m	2012		long QT, VT, TdP	na	na	low K	>700	heroin addiction, hyperthyroidism traces of opioids	[27]
31	f	2012		long QT, TdP	8.75 *	na	low K, Mg	616	alcohol addiction no other medication	[27]
43	f	2012		long QT	na	0.37	low K	480	heroin/benzodiazepin addiction, on methadone methadone	[27]
33	m	2012		long QT, VF	10 *	0.68	na	593	na no cocaine, heroin, methadone > 48 h	[28]
63	m	2012		long QT, VT, TdP	10.5 ^§^	na	low K	498	heroin addiction short-acting opioids ^#^	[29]
young	m	2013		long QT, VT, TdP	17.5	na	na	600	na no alcohol; no heroin, methadone for >72 h	[30]
26	m	2014		long QT, VT, VF	35	0.95	low K, Mg	663	healthy no other drugs	[31]

Notes: na: not available; VF: ventricular fibrillation; TdP: torsade de point; VT: ventricular tachycardia; *: assuming a body weight of 60 kg; #: likely due to patient’s history; §: 10.5 mg/kg of ibogaine HCl (85%–98% purity) and *T. iboga* root extract with 50% alkaloid content; $: abbreviation for ibogaine blood concentrations.

Ibogaine can be extracted from the root bark of *Tabernanthe iboga* which contains about 0.3% ibogaine [32], or by semisynthesis from the precursor compound voacangine, present in *Voacanga africana* at about 0.5%. A total synthesis of ibogaine was first reported by [33], and has improved since then to overcome limitations such as low yield, harsh reaction conditions and bad economics [34]. An efficient strategy for the total synthesis of ibogaine and analogues was reported in 2012, with most reaction steps relying on palladium catalysis [34]. Mass, ^1^H- and ^13^C-NMR spectra are provided in the respective literature [2]. Besides ibogaine (80%), other major components of *Tabernanthe iboga* root bark extracts include ibogaline (15%), ibogamine (up to 5%), and to a lesser extent tabernanthine and voacangine [5].

In the body, ibogaine is metabolized to its main metabolite noribogaine in the gut wall and liver [35]. Ibogaine is O-demethylated to noribogaine primarily by cytochrome P4502D6 (CYP2D6) enzymes. Minor contributions to the metabolism of ibogaine are also provided by CYP2C9 and CYP3A4 [35,36]. CYP2D6 constitutes an important enzyme contributing to the metabolism of approximately 30% of the currently used drugs [37]. Abundant polymorphisms within the CYP2D6 gene account for a complete loss of enzyme function in approximately 5%–10% of the Caucasian population, and is made responsible for significant inter-individual differences in drug metabolism. This may have important implications for drug-drug interactions (see below).

Ibogaine is subject to extensive first pass metabolism as indicated by experiments in rats [38], and by the rapid appearance of the main metabolite, noribogaine [35]. Ibogaine levels peak 2 h after ingestion (oral doses, 500–1000 mg) reaching a free maximum plasma concentration (C_max_) of approximately 1 µg/mL, and the majority of the alkaloid (>90%) is eliminated after 24 h (plasma half-life, 4–7 h) [1,9,35,39]. Consistent with ibogaine’s lipophilic nature the drug accumulates in fat, where its levels become much higher than in plasma or brain [38]. Noribogaine levels (peak, 2.5 h after ingestion; C_max_ ~1 µg/mL), on the other hand, did not significantly decline within 24 h [9,35,39], and the half- life of the metabolite in human plasma was recently estimated being 28–49 h [40]. Solid pharmacokinetic data on human subjects after ibogaine uptake can be found in publications from the group of Mash [9,35,39], who ran a detoxification clinic in St. Kitts, West Indies. Ibogaine concentrations have been measured in whole blood samples of humans after single oral doses (500–1000 mg) [9,39], doses that are typically applied to treat drug addicts (10–25 mg/kg of body weight) [2,9,39], and in a case of ibogaine intoxication [41]. The values determined were 1–10 μg/mL (3–30 μM) and represent total drug concentrations. With the extent of ibogaine's human plasma protein binding of 65% [17] taken into account, the free plasma concentrations reached after drug uptake in these studies amounted to 1–11 μM [17]. In contrast to ibogaine, the mechanisms involved in the metabolism of noribogaine are mostly unresolved. A recent study suggests the involvement of glucuronidation [40], but there is no information regarding CYP- mediated pathways as yet. Finally, for the ibogaine congener 18-MC, the predominant metabolic pathway is 18-hydroxycoronaridine (18-HC) formation, and this is primarily catalysed by CYP2C19 [42].

## 3. Effects on the Cardiovascular System

### 3.1. Cardiac Electrophysiology

Several indole alkaloids exert effects on the cardiovascular system, and have been or are still used as therapeutic drugs. Among those reserpine has a history as antihypertensive drug, and ajmaline is still approved as an antiarrhythmic medicine. Cardiovascular effects of iboga alkaloids have been known for many years. For example, [10] administered ibogaine to cats and dogs and reported a negative chronotropic effect of the drug. Tabernanthine, contained in *Tabernanthe iboga* root bark extracts, induced bradycardia and hypotension when applied to rats and dogs [43,44]. Furthermore, this alkaloid induced a negative inotropic effect in electrically stimulated rat myocardial tissue [45]. In rats, low doses of ibogaine (40 mg/kg) did not change the resting heart rate or the blood pressure. However, at higher doses (100–200 mg/kg), ibogaine decreased the heart rate without affecting blood pressure [4]. In contrast to [4], a significant decrease in heart rate was found in rats already at low ibogaine doses [46]. 18-MC, even at high doses, did not show any apparent effects on either heart rate or blood pressure [4]. Besides these findings on animals, anecdotal evidence suggests that ibogaine can also slow the heart rate in humans [1,6]. Mash *et al.* performed intensive cardiac monitoring in 39 human subjects who received single doses of ibogaine for the treatment of cocain/heroin addiction [47]. With dosages in the range of 500–1000 mg, six out of 39 subjects showed a significant bradycardia, and one subject a significant hypotension.

#### 3.1.1. Ibogaine Effects on the Heart Rate

In order to explain ibogaine’s bradycardic action in animals and humans, several mechanisms have been discussed in the literature. Although none of those mechanisms has been conclusively proven as yet, this complex topic is worth considering in more detail. First, a psychoactive drug modulating several neurotransmitter systems, such as ibogaine, may generate effects on the cardiovascular system related to its central nervous activity. Further, ibogaine was shown to inhibit voltage-gated calcium channels in rat sympathetic and parasympathetic neurons via sigma receptor activation [48]. This may influence cell-to-cell signalling in autonomic ganglia, and thus the regulation of heart rate by the peripheral nervous system. Interestingly, compared to ibogaine, 18-MC shows significantly less affinity to sigma receptors [4,49]. If the heart rate is indeed affected by sigma receptor activation, 18-MC will be less effective in this regard.

Ibogaine’s bradycardic action was often related to stimulatory effects of the drug on the cholinergic system [1,2,4,49]. Here, two possible mechanisms were proposed: (1) inhibition of acetylcholinesterase by the drug; and (2) an agonistic action on muscarinic acetylcholine receptors. Whereas the former potential mechanism could plausibly be ruled out by showing that ibogaine’s inhibition of acetylcholinesterase is physiologically negligible [50], muscarine receptor agonism still remains an option. Thus, ibogaine’s reported affinities for M_1_ and M_2_ muscarinic receptors are in the low micromolar range [2,4,51]. However, a direct functional proof for the proposed assumption that ibogaine is an agonist at muscarinic receptors is lacking as yet. 18-MC’s affinity for muscarinic receptors is at least two-fold less than that of ibogaine [4,49].

A further postulated mechanism by which ibogaine could induce bradycardia is a blockade of voltage-gated sodium channels [1,4,49,50]. Indeed, ibogaine has low micromolar affinity for sodium channels in the brain [49,51,52], and cardiac sodium channel blockers can exert a bradycardic effect [53,54]. We recently tested ibogaine’s effects on human cardiac Na_v_1.5 sodium channels heterologously expressed in TSA-201 cells [17]. Na_v_1.5 is the major sodium channel isoform expressed in the heart [55,56]. To our surprise we found that low micromolar ibogaine (or 18-MC) concentrations did not affect Na_v_1.5 channel currents at all. We reported IC_50_ values for Na_v_1.5 current inhibition by these two drugs >100 µM. Thus, the affinities of both ibogaine and 18-MC for Na_v_1.5 channels seem to be much lower when compared to the affinities of these drugs for brain sodium channels. We concluded that ibogaine is not a considerable sodium current inhibitor in the heart, because the maximum free plasma concentrations reached after drug intake are approximately 10 μM [17]. Consequently, it is unlikely that cardiac sodium channel inhibition by ibogaine significantly contributes to bradycardia generation. Here, however, it is noteworthy to mention recent findings suggesting that besides Na_v_1.5, also brain type sodium channels contribute to the total sodium current in human cardiac myocytes [57]. These brain type channels may indeed be inhibited by low micromolar ibogaine concentrations, and an effect on the heart rate cannot entirely be excluded based on what is known today.

Finally, there are numerous other mechanisms by which a drug like ibogaine could generate bradycardia. In this context, the modulation of ion channels, representing major physiological determinants of the heart rate, deserves special consideration. These are channels which contribute to diastolic depolarisation in pace-maker regions of the heart such as e.g., hyperpolarisation-activated cyclic nucleotide-gated channels, T-type calcium channels, and Ca_v_1.3 L-type calcium channels, as well as G-protein-gated inwardly rectifying potassium channels. The latter channels mediate an outward potassium current, that, when activated by parasympathetic signals such as acetylcholine, slows the heart rate [58]. If ibogaine exerts a considerable modulatory effect on any of the named channels in low micromolar concentrations, alterations in heart rate will result. Although, to our knowledge, such effects of ibogaine have not been described as yet, they are not unlikely to occur considering that ibogaine is a “dirty drug”, interacting with many different molecular targets and receptors in the body.

In summary, there are many potential mechanisms to explain ibogaine’s bradycardic effect in animals and humans. At the moment it is completely unclear which mechanism or which combination of mechanisms essentially contribute. Given the complexity of both the alkaloid’s pharmacology and the regulation of physiological heart rate, it is well conceivable that this issue will never be completely resolved.

#### 3.1.2. Ibogaine Effects on Cardiac Ion Channels

As mentioned in the introduction, cases of ibogaine-induced QT interval prolongation and associated life-threatening TdP arrhythmias e.g., ([21,27,28]) have been accumulating in recent years (Table 1). The most common reasons for QT prolongation and arrhythmia induction by drugs are modulatory effects on cardiac ion channels. It is therefore noteworthy to look at ibogaine’s effects on ion channels with major importance for electrical impulse conduction in the heart.

In this context, we have recently studied the effects of ibogaine and its congener 18-MC on human cardiac hNa_v_1.5 sodium, hCa_v_1.2 L-type calcium, and human ether-a-go-go-related gene (hERG, hK_v_11.1) potassium channels heterologously expressed in TSA- 201 cells [17,59]. We found that low micromolar ibogaine concentrations, resembling the free drug levels reached in human plasma after drug intake [17], did not considerably block both hNa_v_1.5 sodium and hCa_v_1.2 calcium channels. The IC_50_ value for sodium current inhibition by ibogaine was 142 µM, and for 18-MC even less affinity (IC_50_, 464 µM) was observed [17]. hCa_v_1.2 currents were inhibited by ibogaine with an IC_50_ value amounting to 163 µM. These findings suggest that cardiac sodium and calcium currents are not significantly modulated by ibogaine and its congener 18-MC in a regime of doses normally used to treat human addicts.

**Figure 2:**
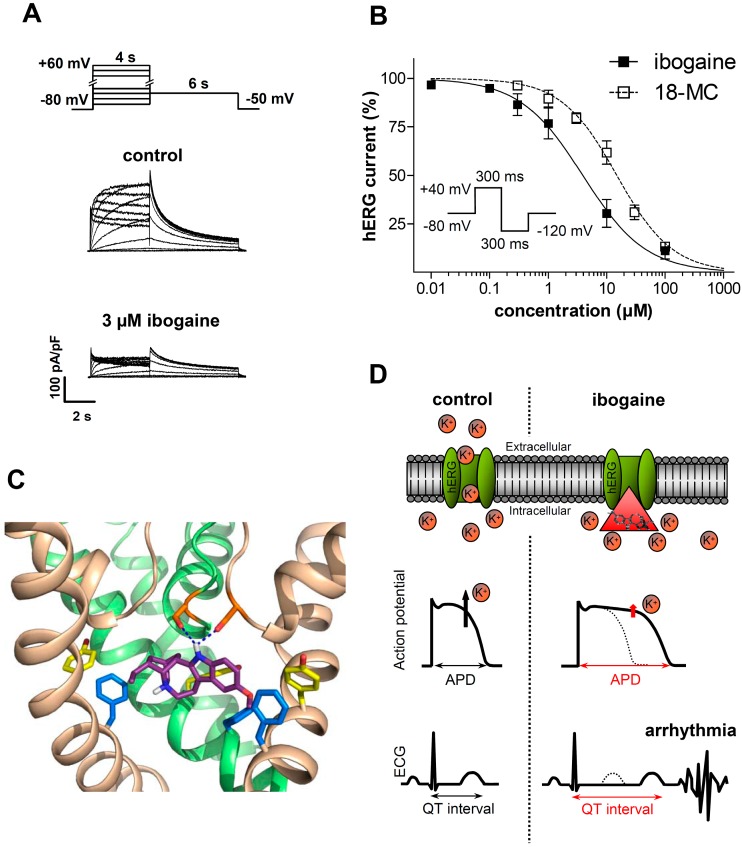
hERG channel inhibition by ibogaine and 18-MC, and its consequences for cardiac electrophysiology. (**A**) Original hERG potassium currents in the absence (control) and presence of ibogaine recorded with the whole cell configuration of the patch clamp technique. hERG channels were heterologously expressed in TSA-201 cells and voltage clamped by the protocol depicted on top. This figure part was taken with permission from [17] (figure 1b) where all experimental details can be found; (**B**) Concentration-response relations for hERG current inhibition by ibogaine (IC_50_, 4 µM) and 18-MC (IC_50_, 15 µM). Currents were elicited by the protocol shown in the inset. Taken with permission from [17] (figure 2c); (**C**) Binding pocket of ibogaine within the hERG channel as revealed by molecular drug docking. hERG inner cavity with flanking S6 helices and pore loops (brown and green). Only three side chains are shown for clarity: residues Tyr652 (yellow) and Phe656 (blue) of the S6 helices and residues Ser624 (orange) on the pore loop as part of the selectivity filter. Ibogaine in its protonated form is shown in purple. Hydrogen bonds are shown as blue dotted lines. Taken with permission from [60] (figure 6); (**D**) Suggested mechanism of cardiac arrhythmia induction by ibogaine: ibogaine blocks hERG potassium channels from the intracellular side (top) and thereby retards repolarization of the ventricular AP (middle). Consequently, the QT interval in the ECG is prolonged (bottom), which finally enhances cardiac arrhythmia risk.

In contrast, when we tested the effects of ibogaine on hERG potassium channels, we found a significant current inhibition already at low micromolar concentrations (IC_50_, 4 µM) [17,59] (Figure 2A,B). The respective IC_50_ value for hERG current inhibition by 18-MC was 15 µM [17] (Figure 2B). In a subsequent study on the mechanism of hERG channel block by ibogaine [60] we could show that the drug passes the membrane to target the channel from the intracellular side. Drug docking and site- directed mutagenesis unravelled the channel’s internal cavity as the site of action; residues of the pore lining S6 segments, as well as adjacent residues of the selectivity filter build up the binding pocket (Figure 2C). The bridgehead nitrogen of ibogaine (see Figure 1) is a tertiary amine with a pKa value of 8.1 [60], causing the drug to co-exist in a charged and a neutral form at physiological pH. Either the charged form alone or in company with the neutral form is responsible for channel block, both species sharing the same binding site and binding pose (Figure 2C). To reach its binding site, ibogaine was contingent on the opening of the channel. Thus, the alkaloid was not able to bind to closed channels, but targeted the open and inactivated conformation of the protein [60].

hERG channels together with K_v_7.1 channels, mediating the IKr and IKs potassium currents in the heart, respectively, are well known as the crucial determinants of the repolarization phase in the human cardiac action potential (AP) [23]. Consequently, their inhibition should induce a prolongation of the cardiac AP. Whereas K_v_7.1 channels were not affected by ibogaine at relevant concentrations (IC_50_ > 100 µM) [60], hERG channels, as reported above, were certainly blocked by therapeutic concentrations of the drug, and an AP prolongation was therefore expected. When testing this hypothesis using a commonly utilized model system for human heart cells, guinea pig ventricular cardiomyocytes [22], we obtained a surprising finding: ibogaine did not prolong, but even shortened the AP at higher concentrations [17]. We could resolve this discrepancy by showing that, in guinea pig cardiomyocytes, contrary to what we found for hCa_v_1.2 channels, ibogaine has a relatively high affinity for L-type calcium channels (IC_50_, 53 µM) [17]. Thus, calcium channel inhibition by the drug obviously counteracts the AP-prolonging effect generated by hERG channel blockade in guinea pig cardiomyocytes. Such a “counteracting effect” may also be present in human cardiomyocytes, where it probably plays a negligible role at clinical concentrations given the low affinity of ibogaine for hCa_v_1.2 calcium channels (IC_50_, 163 µM) [17]. In this respect ibogaine is similar to the alkaloid quinidine, which blocks L-type calcium channels at high concentrations [61], whereas the drug’s hERG channel blockade is observed at low concentrations [62].

Very recent experiments in our research laboratory on human induced pluripotent stem cell-derived ventricular-like cardiomyocytes (unpublished data) indeed confirm our prediction [17] that low micromolar ibogaine concentrations prolong the human cardiac AP. In addition, we find a flattening in AP repolarization in the presence of the drug.

Prolongation of the AP in ventricular cardiomyocytes is thought to directly go along with a prolonged QT interval in the ECG [22,23]. Furthermore, hERG channel blockade by drugs is considered the most prevalent reason for drug-induced QT interval prolongation. We therefore conclude from our electrophysiological studies that ibogaine, in the doses currently used to treat addicts, can induce QT interval prolongation. As mechanism we propose inhibition of hERG potassium channels by the drug. Inhibition of hERG channels will reduce the potassium efflux during repolarization, and will result in an increase in human cardiac AP duration, concomitantly leading to a prolongation of the QT interval (Figure 2D) [22]. Since 18-MC also inhibits hERG channels in low micromolar concentrations [17], it may also prolong the QT interval. 18-MC’s affinity for hERG channels, however, is 3- to 4-fold less than that of ibogaine (Figure 2B), and may thus be less “effective” in the case of similar free drug concentrations in the blood. Further, our findings readily explain previously published case reports of QT interval prolongation after ibogaine intake (e.g., [21,27,28]). The as yet puzzling clinically observed phenomenon that QT prolongation after intake of a single ibogaine dose can last for several days (e.g., [21,27,28]), significantly longer than the half- life of the alkaloid in human plasma (4–7 h) [1], can be resolved by another recent observation in our laboratory. Thus, we find that not only ibogaine, but also its long-lived active metabolite noribogaine (half-life, 28–49 h [40]) inhibits hERG channels and prolongs the human cardiac AP at low micromolar concentrations (unpublished data). Very recently [63] reported an IC_50_ value of 3 µM for hERG channel inhibition by noribogaine, which is consistent with our data.

Here a further point should be taken into consideration. It is a well-known phenomenon that many drugs targeting hERG channels do not only exert direct modulatory action on channel function, but also an indirect effect by influencing channel trafficking, *i.e*., the normal folding and protein export from the ER to the plasma membrane. Via this indirect influence functional channel expression at the cell surface can be inhibited or boosted, and thus result in decreased or increased channel availability, respectively. In a recent article screening numerous hERG channel blockers 40% of the drugs tested positive for additional impairment of hERG protein trafficking [64]. If ibogaine, noribogaine, and/or 18-MC really influence hERG trafficking remains to be determined. It is tempting to speculate that ibogaine-induced inhibition of hERG channel trafficking is involved in the observed long-term prolongation of the QT interval after ibogaine application.

Finally, the described effects of ibogaine and noribogaine: prolongation and flattening of the human cardiac AP (unpublished data), and relative selective hERG channel inhibition [17,59,63] as triggers of QT interval prolongation must be considered proarrhythmic. Here we want to emphasize that not only QT prolongation itself [22,23], but also and especially a flattening of the repolarisation phase in the AP is considered a proarrhythmic characteristic [22,65]. Moreover, ibogaine’s effects on *in vitro* cardiac electrophysiology within the drug’s therapeutic plasma concentration range, closely resemble the cardiac actions of formerly approved drugs like cisapride or astemizole, which are known to be unsafe, and have been withdrawn from the market because of their pronounced propensity to induce TdP arrhythmias [22]. Thus, ibogaine, at the doses currently used in humans, must be classified an unsafe drug! Because of its considerably longer half- life in human plasma, ibogaine’s metabolite noribogaine, rather than the parent drug itself, might represent the major proarrhythmic factor. This clearly challenges the concept of noribogaine being considered a potentially safer alternative to ibogaine for anti-addiction medication development [6,7,8].

### 3.2. Clinical Evidence of Cardiotoxicity

#### 3.2.1. Clinical Reports

Preclinical toxicological testing in dogs on behalf of NIDA did not indicate prolongation of the QT interval [1], and no abnormalities were observed in the ECG under continuous cardiac monitoring in 39 subjects receiving single doses of ibogaine for the treatment of cocain/heroin addiction (dosage between 500 and 1000 mg) [47]. However, between the years 1990 and 2008 a total of 19 fatalities temporally associated with the ingestion of ibogaine were reported (reviewed in [1]) from which six subjects died of acute heart failure or cardiopulmonary arrest. Moreover, since then additional reports of sudden death turned up, and numerous cases of QT interval prolongation in the electrocardiogram accumulated. This new evidence not only suggests a causal relationship between the intake of ibogaine and a prolongation of the QT interval, but also points out the drug-associated potential to develop dangerous ventricular tachycardias including the emergence of life-threatening TdP arrhythmias. The following section provides a summary of all recent (2009–2014) episodes of dangerous QT interval prolongations and ibogaine-associated cardiac arrhythmias. We further summarize all recent case reports of fatalities, and compare them with the compilation of older cases provided by [1].

Ingestion of ibogaine has been temporally related to several cases of sudden death. A systematic overview of all reported cases in the years between 1990 and 2008 is given by [1] (Table 1: first line). The authors list and describe a total of 19 fatalities that occurred after ibogaine administration, mostly for reasons of opioid detoxification or treatment of alcohol dependence. In 14 of these cases adequate *post-mortem* data existed which demonstrated that almost all of the decedents suffered from pre-existing medical conditions or were compromised by the additional intake of other drugs such as opiates and cocaine [1,6]. The predominant part of concomitant autopsy findings were of cardiovascular nature: coronary artery sclerosis, hypertension, myocardial infarct, cardiac hypertrophy, and dilated cardiomyopathy, but also hepatitis, liver cirrhosis and steatosis are mentioned [1]. If systemic disease is a confounding factor that contributes to the morbidity of ibogaine, or even constitutes a prerequisite for the occurrence of fatalities, remains to be resolved. Interestingly, no signs of neurotoxicity were found in these autopsies [1], as opposed to the results from earlier experiments on animals (e.g., [18,19,20]).

Since 2008, an additional three cases of ibogaine- ascribed sudden death have been reported (Table 1) [24,25,26]. The case of a 52 year old man with a 20 year history of alcoholism and previous episodes of hypokalemia and hypomagnesemia who died suddenly upon intake of ibogaine was described in [24]. Post mortem examination revealed sclerosis of the coronary arteries and liver cirrhosis and steatosis as possible concomitant morbidity. [25] described the case of a 27 year old caucasian male with a 15 year history of multiple substance abuse but no underlying cardiac pathology. He received a methadone-based substitution treatment and was additionally medicated with diazepam. The forensic scientist stated an ibogaine-borne mixed overdose in association with methadone and diazepam. The case of a 25 year old man with history of supraventricular tachycardia and chronic heroin addiction was presented in [26]. The man experienced muscle spasms and ataxia and developed respiratory difficulties and cardiopulmonary arrest. Despite successful resuscitation the patient deceased after 2 days because of multi-organ failure. Taken together, these new cases of ibogaine-induced deaths resemble those described in [1], in the existence of a cardiac predisposition and/or concomitant intake of other drugs as possible contributing factors of morbidity.

Besides these fatalities, several case reports have accumulated recently describing a prolongation of the QT interval in the ECG after intake of ibogaine (Table 1, lower block). As the QT interval displays a significant dependency on the heart rate, this ECG parameter is often reported in a corrected form. A heart rate corrected QT interval (QT_c_) of >470 and >450 ms was associated with an increased risk of sudden death, for women and men, respectively, and a QT or QT_c_ interval exceeding 500 ms can be regarded a significant risk for TdP [66,67,68]. A total of eight such cases have been described since 2008, affecting five male and three female subjects in the age of 26–63 years [21,27,28,29,30,31]. The reported QT_c_ values ranged from minor prolongation of only 480 ms to a dramatic one with more than 700 ms in duration. A shortcoming being worth a note here is that all these case reports lack the indication of the actual method used for heart rate correction of the QT interval. Ibogaine’s effect on the heart rate, together with the use of an inadequate correction method, may have therefore confounded QT_c_ value calculations in some cases. All subjects took ibogaine for treatment of their alcohol or heroin addiction except for one individual (described in [31]) seeking spiritual experience. Except of one case (see Table 1) the prolongation of the QT interval subsequently evolved into ventricular tachycardia (VT) and/or ventricular fibrillation (VF). In the majority of cases a subsequent emergence of TdP arrhythmias was observed [27,29,30]. Only in one case the lack of arrhythmia occurrence was reported [27]. However, in this case the QT interval was only minimally prolonged (480 ms; Table 1, line 8 from above). In all instances proper medical interventions likely saved the lives of these people.

Noteworthy is that ibogaine intake is typically not immediately accompanied by deleterious adverse events. As can be seen from Table 1, the ibogaine-ascribed deaths occurred between 1.5–76 and 12–24 h after drug intake, in the older [1] and the newly reported (Table 1) cases, respectively. Considering that ibogaine has a half-life of only 4–7 h in human plasma [1] the appearance of fatalities 24–76 h after drug ingestion can hardly be attributed to the sole action of the alkaloid. Moreover, QT interval prolongation after ibogaine administration typically lasts for more than 24 hours [21,27,28,29,31], and has been observed to sometimes persist for longer than a week [27,28]. Hence, it seems plausible that ibogaine’s long-lived metabolite noribogaine, rather than the parent drug itself, constitutes the major cardiac risk after ibogaine intake.

#### 3.2.2. Risk Factors

Based on the findings of the above described experimental studies and case reports on ibogaine’s cardiovascular actions, this section deals with risk factors and their practical impacts for the future application of ibogaine and 18-MC as anti-addiction drugs.

First, application of ibogaine, by trained medical personnel only, should be permitted merely under strict medical observation and continuous electrocardiographic monitoring for an extended time period, which carefully takes noribogaine’s longevity in human plasma into account.

Secondly, prior to ibogaine application one should carefully consider additional risk factors for drug-induced TdP arrhythmias in a patient including female gender, a prolonged baseline QT interval, bradycardia, abnormal electrolyte levels, preexisting cardiovascular disease, ion channel (e.g., hERG) mutations, drug-drug interactions, and genetic variants influencing drug metabolism [22,69,70,71]. For anti-addiction treatment with ibogaine, two among the named risk factors deserve special attention: bradycardia and hypokalemia. Thus, ibogaine itself induces bradycardia, and hypokalemia is frequent in drug users (see below). When related to the large number of people who have received ibogaine treatments over many years worldwide, comparably few fatality cases have occurred or have been reported [1] (Table 1). In addition, drug safety studies on human addicts performed under well-controlled conditions revealed no significant adverse effects [35]. Here baseline screening included a medical evaluation, physical examination, ECG recording, blood chemistries, and haematological workup, as well as psychiatric and chemical dependency evaluations. In some cases more extensive evaluations were performed to rule out cardiac risk factors and to exclude subjects for study entry. Cardiac monitoring demonstrated that no electrocardiographic abnormalities were produced or exaggerated following ibogaine administration in subjects that were not comorbid for any cardiovascular risk factors [35]. Here, however, it is possible that more sensitive and sophisticated methods to detect QT_c_ changes and arrhythmias, as those available nowadays, might have exposed abnormalities. Together, these results suggested that, in principle, ibogaine is well tolerated in drug-dependent subjects. As a consequence, this implies that not ibogaine application alone, but rather the drug in connection with one or more additional risk factors induces arrhythmias and sometimes sudden cardiac death. Some of these “second hit” risk factors, which can bring out ibogaine’s cardiotoxic potential, are considered in more detail in the following:

(i) Altered Electrolyte Levels: Hypokalemia and Hypomagnesemia

In all case reports of ibogaine associated fatalities and cardiac arrhythmias (Table 1) hypokalemia was detectable when tested. Potassium levels, normally around 3.5–5 mM, were found as low as 2 mM [31], which reflects a severe hypokalemic condition [72]. The frequently altered nutritional status of substance abusers, in particular regarding alcohol abuse, may provide one explanation for this observation [24]. Although hypokalemia is common and may be found in up to 20% of hospitalized people [72], the observed incidence (100%) is striking and suggests that this condition predisposes for ibogaine cardiotoxicity. Indeed, hypokalemia has a two-fold implication in generating long QT and an increased risk of TdP. First, lowering of extracellular potassium directly decreases hERG currents in the heart [71,73,74]. Secondly, low extracellular potassium can also increase the blockade of hERG currents by drugs [70,71]. Preliminary experiments in our laboratory do in fact suggest that also hERG channel inhibition by ibogaine is reinforced under low external potassium (unpublished data). We conclude that hypokalemia very likely enhances QT prolongation and the TdP risk associated with ibogaine ingestion via boosting the direct hERG channel inhibitory effects of ibogaine and noribogaine.

Hypomagnesemia was present in 50% of the case reports described in Table 1, and was always accompanied by hypokalemia. It is believed that also hypomagnesemia increases TdP risk [71]. The underlying mechanism, however, is less clear, and probably involves the modulation of L-type calcium channel function, which may contribute to the development of early after depolarisations [71]. Thereby, the occurrence of hypomagnesemia may reinforce ibogaine’s cardiotoxic potential, particularly dangerous in the case of simultaneous incidence of hypokalemia.

(ii) Drug-Drug Interactions and Genetic Polymorphisms

Addicts often have a long history of substance abuse. Alcohol, heroin, cocaine, benzodiazepines, and methadone are among the abused substances, frequently also combinations thereof. Methadone is also prescribed for opioid substitution therapy. When ibogaine is administered to addicts, the presence of other substances (prescribed and/or illicit) in the patient’s blood plasma is therefore not uncommon [1] (Table 1). This paves the way for adverse drug interactions. Concerning the heart, several substances of abuse have been associated with hERG channel inhibition and/or QT interval prolongation, e.g., alcohol [1,75], cocaine [1,76], and methadone [1,77,78]. If relevant concentrations of such substances are still residing in the plasma when ibogaine is applied, the drug’s QT prolonging effect, and thus the risk of TdP arrhythmias will be raised. Here, methadone deserves special attention because it additionally has an exceptionally long plasma half- life (15–55 h) [79].

Another possibility for drug-drug interactions to influence the cardiotoxic potential of ibogaine is impairment of the drug’s metabolism. Ibogaine is metabolised to noribogaine mainly by CYP2D6 (see above). This enzymatic transformation can easily be affected by drugs that are metabolised via the same route leading to saturation of the system, or by direct interaction of the drug with the metabolising enzyme, as e.g., observed for methadone which inhibits CYP2D6 [80]. It is thus likely that relevant plasma levels of methadone will impair the pharmacokinetics of ibogaine. Since ibogaine’s active metabolite noribogaine has very similar cardiotoxic potential than the parent drug itself (see above), also drugs affecting noribogaine metabolism will be of relevance. Here speculations about specific drug-drug interactions are impossible because noribogaine’s metabolism is basically unsettled (see above). Preconceiving that noribogaine *per se* has a long half-life in human plasma in the order of 1–2 days [40], the specific inhibition of its metabolism by other drugs will have strong impacts. Thus, in such a case, cardiotoxic effects may even persist for weeks after intake of a single dose of ibogaine. Therefore, we strongly encourage researchers to determine the pathways involved in the metabolic conversion of noribogaine. Finally, 18-MC’s cardiotoxicity will be altered by drugs that modulate the function, or are a substrate of CYP2C19, because 18-MC is mainly metabolised by this particular CYP enzyme [42].

Besides drug-drug interactions modulating the cardiotoxic potential of ibogaine, noribogaine, and 18-MC, there are also genetic polymorphisms that need to be considered. In example, with relevance for ibogaine, extensive genetic polymorphisms within the CYP2D6 gene have been described [37], which lead to substantial variations in inter- individual enzyme activities. Depending on their allelic variants people can be divided into different classes of metabolisers: ultra-rapid, extensive, intermediate, and poor [37]. In terms of ibogaine, poor metabolizers showed an increased latency to reach maximum plasma concentrations (C_max_) of the drug, as well as greatly diminished noribogaine C_max_ levels [35]. Obviously, if as yet unknown genetic polymorphisms exist, which generate poor noribogaine metabolizers, these will also be of great relevance for ibogaine application. Thus, in poor noribogaine metabolizers, a single ibogaine dose may suffice to induce cardiotoxicity persisting for weeks. Finally, genetic polymorphisms within the CYP2C19 gene [81] should be considered in case of 18-MC application.

Is the Ibogaine Congener 18-MC Less Cardiotoxic than Ibogaine?

In contrast to ibogaine, even high doses of 18-MC did not show any apparent effects on either heart rate or blood pressure in animal experiments [4]. We have previously shown that the affinity of 18-MC to human cardiac voltage-gated ion channels is lower than that of ibogaine [17]. In principle, these findings imply a reduced risk for cardiac adverse effects for 18-MC in comparison with ibogaine application. However, 18-MC’s affinity for hERG channels (IC_50_, 15 μM) is still near to the therapeutic concentration range (see above), if similar plasma protein binding as for ibogaine is assumed. Thus, like ibogaine, 18-MC may have the propensity to induce QT interval prolongation and TdP arrhythmias. A recent study from Alper’s group [63] also suggested that 18-MC shows lower affinity for hERG channels than ibogaine. Here, the IC_50_ for hERG current inhibition was estimated to lie between 50 and 100 µM, a value considerably higher than we reported in [17]. Together, these studies suggest that, compared with ibogaine, 18-MC is less cardiotoxic, and has a lower propensity to induce TdP arrhythmias when applied in similar concentrations. The lack of knowledge about the amount of 18-MC’s plasma protein binding, however, is an element of uncertainty which remains.

## 4. Conclusions

The anti-addiction drug ibogaine certainly affects the heart and the cardiovascular system. Besides lowering the heart rate, therapeutic concentrations of the alkaloid interact with cardiac ion channels, and these effects most likely determine ibogaine’s potentially life-threatening cardiotoxicity. Based on the findings of the above described studies, we propose the following sequence of deleterious events to explain the cardiotoxicity associated with the application of ibogaine as anti-addiction drug (Figure 2D): (1) blockade of repolarizing hERG potassium channels; (2) retardation of the repolarization phase of the ventricular AP; and (3) concomitant prolongation of the QT interval in the ECG, ultimately paving the way for life-threatening TdP arrhythmias. Due to the longevity of noribogaine—ibogaine’s active metabolite—in human plasma, cardiac adverse events may also occur several days, in some cases weeks after intake of a single dose of ibogaine. Noribogaine, on the other hand, may also convey long-lasting anti-addictive efficacy after ibogaine application.

Future administration of ibogaine to treat addiction may be justified by the urgent medical need for an effective anti-addiction drug. Thus, the use of a drug known to prolong the QT interval must be based on risk-benefit analysis in individual patients. Where benefit outweighs risk, QT prolongation should not limit necessary therapy [82]. Recommendations on prevention and guidelines for the management of drug-induced QT prolongation and TdP in hospital settings can be found in [82]. Two things need to be carefully considered: (1) ibogaine application should only take place under strict medical observation and continuous electrocardiographic monitoring over a sufficiently long period of time; and (2) additional risk factors for drug-induced TdP arrhythmias must be clarified prior to ibogaine application. Eventually, informed consent should be received from drug addicts, in which the risk of ibogaine-induced sudden cardiac death is appropriately highlighted. Based on the rapidly accumulating knowledge about the potentially harmful cardiotoxicity of ibogaine, the responsible medical authorities are now requested to define respective standards and exclusion criteria to allow for a safer ibogaine anti-addiction therapy in the future.

Finally, the ibogaine congener 18-MC is likely associated with a reduced risk of TdP arrhythmia induction, because it shows considerably lower affinity for hERG channels than ibogaine. We encourage researchers to develop ibogaine-like drugs with preserved anti-addictive properties, but negligible hERG affinity, and thus absent TdP arrhythmia risk.
